# Generating the Critical Ising Model via SRGAN: A Schramm–Loewner Evolution Analysis from a Geometric Deep Learning Perspective

**DOI:** 10.3390/e28040385

**Published:** 2026-03-31

**Authors:** Yuxiang Yang, Wei Li, Yanyang Wang, Zhihang Liu, Kui Tuo

**Affiliations:** 1 Key Laboratory of Quark and Lepton Physics (MOE) and Institute of Particle Physics, Central China Normal University, Wuhan 430079, China; yuxyang@mails.ccnu.edu.cn (Y.Y.); zhliu123@outlook.com (Z.L.); tuokui@mails.ccnu.edu.cn (K.T.); 2 SCIQ Lab, ESIEA, Campus Ivry sur Seine, 73 bis Avenue Maurice Thorez, 94200 Ivry sur Seine, France; 3 Wuhan Technology and Business University, Wuhan 430065, China

**Keywords:** critical ising model, Schramm-Lowner evolution, SRGAN

## Abstract

The geometric signatures of macroscopic interfaces in the two-dimensional critical Ising model strictly adhere to Schramm–Loewner Evolution (SLE) theory. In this study, we propose a physics-driven generative approach using Super-Resolution Generative Adversarial Networks (SRGANs) to approximate the inverse coarse-graining operation to generate larger configurations. From the perspective of Geometric Deep Learning (GDL), we leverage the geometric priors of Convolutional Neural Networks (CNNs)—specifically their translational and rotational symmetries—to effectively encode the universal physical laws of the Ising Hamiltonian. This inductive bias allows the model to be trained on small scales yet be generalized to large-scale systems (2048 × 2048) while preserving physical conservation. To accommodate spin discreteness, we employ an L1-based loss function to maintain domain wall sharpness. SLE analysis and long-range correlation functions confirm that the model reproduces critical dynamics and conformal invariance, successfully serving as a physics-preserving inverse coarse-graining transformation framework.

## 1. Introduction

The two-dimensional Ising model, as the prototype of ferromagnetic phase transitions, serves as the bedrock for understanding critical phenomena. As the temperature *T* approaches the critical point $T_{c}$, the interface length increases dramatically, structures become increasingly intricate, and spin fluctuations exhibit long-range correlations. Consequently, macroscopic interfaces display non-trivial fractal geometries. While local fluctuations in these structures hint at universal statistical laws, their precise mathematical description and deep connection to the system’s physical attributes have long been a subject of intense scrutiny [1,2]. The interface structure in Ising systems is more than a geometric boundary distinguishing phases; it is a fundamental statistical mechanics problem linking the system’s Hamiltonian to configuration probabilities [3,4,5,6].

A breakthrough in this domain came with Schramm’s Stochastic Loewner Evolution (SLE) theory [7,8]. SLE defines a family of conformal maps determined by Loewner differential equations coupled with Wiener processes, where the geometric universality class is governed by a single diffusion coefficient κ. Mathematically, it has been proven that in the continuum limit, the interface of the 2D critical Ising model strictly converges to an SLE curve with κ=3 [9,10]. Within this framework, local correlations and scale invariance are no longer abstract concepts but are concretely encoded as the Brownian motion characteristics of the driving function. This makes SLE not just a theoretical benchmark for describing critical geometry, but a powerful criterion for testing whether numerical simulations or generative models have truly captured the correct topological and geometric features.

Deep learning has found widespread application in phase transition models [11,12] recently, ranging from identifying transition points to phase classification. This has established a new paradigm: validating machine learning algorithms on systems with explicit Hamiltonians, then extending them to systems where the Hamiltonian is unknown [13,14,15,16,17]. A natural question arises in statistical physics: Can we use neural networks to learn the inverse coarse-graining transformation, thereby generating large-scale critical configurations at low cost [18]? This physical super-resolution is far more complex than standard image processing—it requires the generated configurations to be not only visually coherent but to strictly obey physical conservation laws, particularly the scaling behaviors prescribed by SLE theory.

Prior work has explored CNN-based super-resolution for lattice spin systems, demonstrating that supervised deep convolutional networks can successfully reproduce thermodynamic observables such as magnetization and specific heat [19,20]. However, when the training objective minimizes only pixel-wise differences between reconstructed and target configurations, the network is implicitly optimizing for the average over all plausible microstates consistent with the low-resolution input—producing configurations that are accurate in a pixel-wise sense but systematically biased away from the true physical distribution. This is an inherent consequence of the objective function itself, independent of network depth or architectural complexity [21]. Generative Adversarial Networks (GANs) [22,23] offer a principled framework to address this limitation: rather than minimizing a fixed pixel-wise loss, the adversarial training objective directly penalizes deviations of the generated distribution from the true data distribution, thereby encouraging the model to reproduce the full statistical structure of the target ensemble [21]. For thermodynamic quantities, which are themselves statistical averages, this bias may be tolerable. For the domain-wall interfaces of the 2D critical Ising model, however, conformal invariance is a property of the full statistical distribution of interface geometries, requiring that scale-invariant fluctuations and long-range correlations be faithfully reproduced at the level of individual configurations, not merely on average. Motivated by this consideration, we explore the application of SRGAN [24], which supplements the reconstruction objective with an adversarial loss proportional to the statistical divergence between the generated distribution $P_{\hat{X}}$ and the true data distribution $P_{X}$. Rather than approximating the pixel-wise mean of plausible configurations, the adversarial objective enforces that the generated microstates align with the true physical ensemble in their overall statistical distribution, which is essential for preserving the conformally invariant structure of critical configurations.

From the perspective of Geometric Deep Learning (GDL), Convolutional Neural Networks (CNNs) inherently possess translational equivariance and locality. These properties share a profound mathematical isomorphism with the Hamiltonian of the Ising model defined on Euclidean lattices [25,26]. And the architecture of the generator in SRGAN incorporates a multi-layer Convolutional Neural Network. We aim to explore whether this architectural geometric prior can help SRGAN cross scales, inferring the critical features of large systems from small, easily simulated ones.

We utilize a model trained on a 512×512 lattice to super-resolve coarse-grained configurations of various sizes. By employing the Vertical Slit Map algorithm to extract the Loewner driving function, we quantify the physical fidelity of the generated configurations through multiple dimensions: the diffusion coefficient κ, fractal dimension, and Left Passage Probability (LPP). The results are compelling: SRGAN not only reconstructs critical interfaces but exhibits surprising scale generalization capabilities—a simplified consequence of its fully convolutional architecture internalizing the locality of physical laws and implicitly learning the scale invariance described by SLE [27,28].

The structure of this paper is as follows: In Section 2, the interface of the Ising model, the structure of our SRGAN, SLE, and the corresponding quantities are introduced briefly. Section 3 shows the validation of training performance and the scale generalization and the fractal features of the configurations generated by SRGAN. In Section 4, we analyze the difference between the MC configurations and SRGAN configurations. Section 5 concludes this paper.

## 2. Model and Numerical Methods

### 2.1. The Interface of the 2D Ising System

We investigate the 2D critical Ising system on a square lattice, with the Hamiltonian defined as

(1) $$H=-J\sum_{\langle ij\rangle }\sigma_{i}\sigma_{j}.$$

where $\sigma_{i}$ and $\sigma_{j}$ represent a pair of nearest-neighbor spins, and J>0 is the exchange interaction constant (set to J=1 in this work). The spin variable $\sigma_{i}\in \left\{-1,+1\right\}$. This model undergoes a second-order phase transition at the critical temperature $T_{c}=2/\ln\left(1+\sqrt{2}\right)\approx 2.269$ [29].

To obtain interface configurations at the critical state, we employ the Metropolis–Hastings algorithm at $T_{c}$ [30]. System sizes L×L are selected as 64×64,128×128, 256×256, 512×512 and 1024×1024 for comparison. Simulations start from a random initial state (high-temperature limit) and run for at least $10^{4}\times L^{2}$ Monte Carlo steps to ensure that thermal equilibrium is reached. To guarantee statistical independence of the interface, we generated 10,000 configurations by running 10,000 separate Monte Carlo simulations for each size. Each simulation starts and evolves independently.

**Figure 1:**
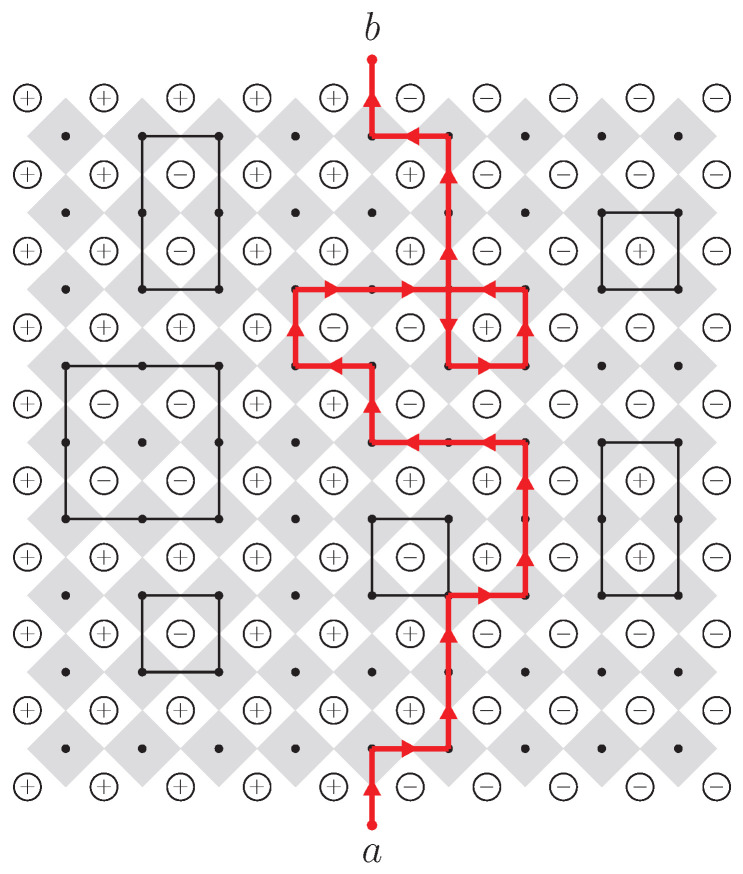
An exploration process to extract the interface curve from the the dual lattice of the Ising configuration, starting from the bottom edge and following the boundary between +1 and −1 spins until we reach the top edge.

**Figure 2:**
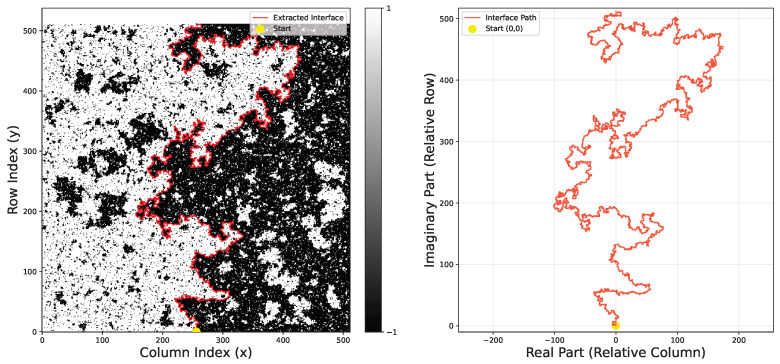
(**Left**) A configuration of the 2D Ising model on a square lattice with Dobrushin boundary conditions, clearly showing an interface connecting the top and bottom boundaries. (**Right**) The interface curve mapped onto the upper half-plane. We use the coordinates of this curve as the subject of our subsequent analysis.

We adopt Dobrushin boundary conditions to induce a single macroscopic interface: the left boundary and the left halves of the top and bottom boundaries are fixed to +1, while the remaining boundaries are fixed to −1. The interface is extracted on the dual lattice, whose sites are located at the centers of the elementary squares of the original spin lattice. As illustrated in Figure 1, the interface is defined as the unique path on the dual lattice that separates +1 spins (denoted ⊕) from −1 spins (denoted ⊖), such that every edge traversed by the path has a +1 spin on its left and a −1 spin on its right. Under Dobrushin boundary conditions, this interface connects a fixed starting point at the midpoint of the lower boundary to a fixed endpoint at the upper boundary, as shown in Figure 2. We map the interface vertices to the upper half complex plane H as the object of analysis for chordal SLE.

### 2.2. Method of SRGAN

**Figure 3:**
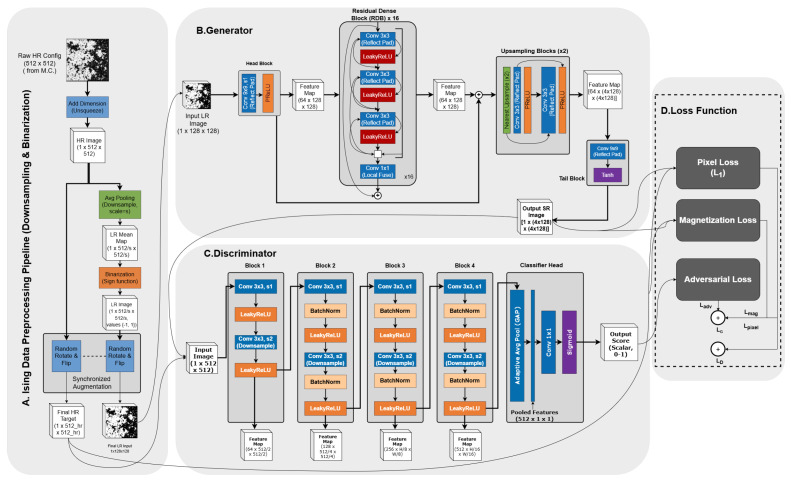
The architecture of our SRGAN. It consists of three parts: Ising configuration preprocessing, the generator, and the discriminator. The generator utilizes Residual Dense Blocks (RDBs) with reflection padding to maintain boundary continuity, upsampling low-resolution Ising configurations to high resolution. The discriminator distinguishes between real MC samples and generated super-resolved samples. The total loss function includes a magnetization constraint ($L_{mag}$), pixel loss, and adversarial loss.

The generator designed for this study adopts a Fully Convolutional Network (FCN) architecture, as illustrated in Figure 3. Unlike traditional classification networks, we completely abandon Dense Layers that are sensitive to input size. This implies that the parameters learned by the model are not tied to pixels at specific locations, but rather represent a set of local filters (convolution kernels) with translational invariance. This sliding window convolution operation physically aligns with the local nature of the Ising model, where spin flips depend solely on their nearest-neighbor environment. Theoretically, convolution kernels trained on small scales can be directly generalized to large-scale systems without adjusting the network structure or retraining. The network includes a fixed ×4 upsampling module (composed of two ×2 PixelShuffle layers), locking the output size to four times the input ($L_{out}=4\times L_{in}$), though the input itself can be a 2D matrix of any size.

Traditional super-resolution methods typically optimize pixel-level Mean Squared Error (MSE). However, MSE essentially averages errors smoothly. While acceptable for natural images, in spin systems requiring binary discreteness, this leads to generated images with numerous non-physical gray intermediate states (blurring) [20]. To counter this, we employ the L1 norm as the content loss because it is more robust to outliers and forces the network to make decisive black or white predictions, thereby maximizing the preservation of domain wall geometric sharpness. The total loss function is defined as

(2) $$L_{G}=\lambda_{pixel}L_{pixel}^{\left(L1\right)}+\lambda_{adv}L_{adv}+\lambda_{mag}L_{mag}.$$

In addition to the L1 content loss ($L_{pixel}$), $L_{mag}$ penalizes deviations in total magnetization between the generated and target configurations, forcing the network to learn the correct order parameter statistics. The generator and discriminator are optimized using the Adam optimizer.

### 2.3. Schramm–Loewner Evolution

Schramm–Loewner Evolution (SLE) describes a family of conformally invariant stochastic fractal curves defined on the complex plane. The evolution of SLE_*κ*_ is governed by the Loewner differential equation [7,31]:

(3) $$\frac{\partial gt(z)}{\partial t}=\frac{2}{g_{t}\left(z\right)-\xi_{t}},\;g_{0}\left(z\right)=z,$$

where $g_{t}\left(z\right)$ is the unique conformal map mapping the upper half-plane minus the curve portion H∖γ[0,t] back to the upper half-plane H, satisfying the hydrodynamic normalization $g_{t}\left(z\right)=z+2t/z+o\left(1/z\right)$. Here, *t* is the Loewner capacity. The central tenet of SLE theory is that if the ensemble of curves γ satisfies conformal invariance and the Domain Markov Property, the driving function $\xi_{t}$ must be a one-dimensional Brownian motion, i.e., $\xi_{t}=\sqrt{\kappa }B_{t}$, where $B_{t}$ is standard Brownian motion and κ is the unique diffusion coefficient.

### 2.4. Calculation of Driving Function

We investigate the inverse problem of SLE: extracting the driving function U(t) from discrete interface coordinates $\left\{z_{0},z_{1},\ldots ,z_{N}\right\}$ [32,33,34]. We employ the Vertical Slit Map algorithm for iterative encoding [35]. For the *k*-th vertex on the interface, we find a conformal map $g_{k}$,

(4) $$g_{t}\left(z\right)=\sqrt{{\left(z-\Delta \xi_{t}\right)}^{2}+4\Delta t},z\in H,$$

which maps the previous tip back to the real axis. Numerically, this is typically implemented by applying the slit map in reverse. Let $z_{k}=x_{k}+iy_{k}$ be the coordinate of the *k*-th vertex after the previous k−1 mappings; the mapping parameters for step *k* are given by

- Driving function increment: $\Delta \xi_{k}=x_{k}$;
- Capacity increment: $\Delta t_{k}=y_{k}^{2}/4$.

Through the iterative formula $w_{k}=g_{k-1}\circ \cdots \circ g_{1}\left(z_{k}\right)$, where $g_{i}$ maps the *i*-th curve segment, we zip the entire curve back onto the real axis. The cumulative driving function and capacity are [33,35]

(5) $$\;U\left(t_{k}\right)=\sum_{i=1}^{k}\Delta \xi_{k},\;t_{k}=\sum_{i=1}^{k}\Delta t_{i}.$$

### 2.5. Fractal Dimension

A fundamental geometric property of the critical Ising interface is its fractal dimension $d_{f}$, which serves as an important independent indicator for verifying the SLE universality class. According to SLE theory, an analytic relationship exists between the fractal dimension and the diffusion coefficient κ: $d_{f}=1+\kappa /8$ [36]. For the Ising model (κ=3), the theoretical prediction is $d_{f}=1.375$. To estimate $d_{f}$ numerically from discrete lattice data, we use Finite-Size Scaling (FSS) analysis. For a fractal curve discretized on a finite lattice with lattice constant *a* and linear size *L*, the total length *S* of the curve traversing the lattice follows a power law as a→0 or L→∞ [37]:

(6) $$S∼a^{1-d_{f}}L^{d_{f}}.$$

### 2.6. Left Passage Probability (LPP)

Beyond local fractal features, SLE theory provides rigorous probabilistic predictions for the curve’s global topological behavior. A powerful criterion for verifying the conformal invariance of Ising interfaces is the Left Passage Probability (LPP). According to SLE theory, for a chordal SLE_*κ*_ curve γ in the upper half-plane H starting from the origin and tending to infinity, the probability P(θ) that the curve passes to the left of any point $z=re^{i\theta }$ is scale-invariant. This implies the probability depends only on the polar angle θ∈[0,π] and is independent of the modulus *r*. Schramm derived the exact analytic formula for this probability [38,39]:

(7) $$P\left(\theta \right)=\frac{1}{2}+\frac{\Gamma (4/\kappa )}{\sqrt{\pi }\Gamma \left(\frac{8-\kappa }{2\kappa }\right)}\cot{\left(\theta \right)}_{2}F_{1}\left(\frac{1}{2},\frac{4}{\kappa };\frac{3}{2};-\cot^{2}\theta \right),$$

where Γ is the Gamma function and _2_*F*_1_ is the Gaussian hypergeometric function. For the critical Ising model (κ=3), this formula provides the form of the theoretical probability distribution curve.

## 3. Results

### 3.1. Validation of Training Performance

We first evaluate the model’s performance at the training size (512×512). We split 10,000 Ising configurations of size 512×512 into a training set (8000), a test set (1000), and a validation set (1000). After warm-up and training, we save the neural network weights that minimized the generator loss function. We then feed 128×128 Ising configurations generated by Monte Carlo simulation into the generator, invoking the saved weights to produce new 512×512 configurations.

**Figure 4:**
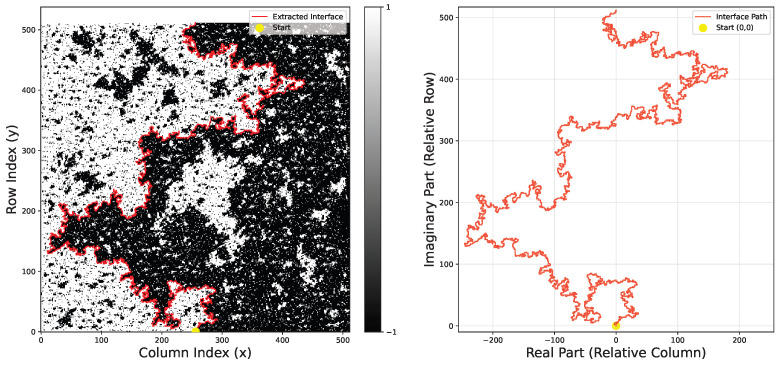
Visualization of Super-Resolution Reconstruction. Low-resolution Ising configurations (128×128) are fed into the trained SRGAN generator, producing high-resolution outputs (512×512). The generated interface (highlighted in red) exhibits complex geometric fractal structures, visually appearing very close to real critical fluctuations.

**Figure 5:**
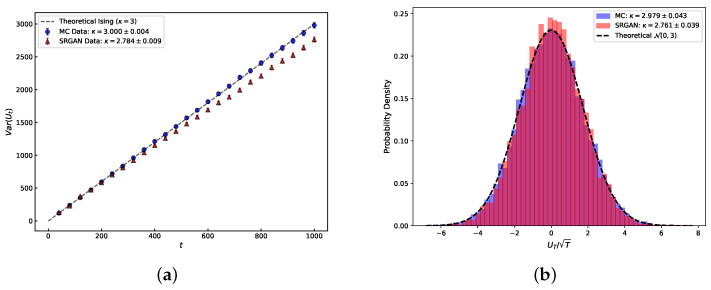
Estimation of the SLE diffusion coefficient κ. (**a**) The variance of the driving function $\mathrm{Var}(U_{t})$ as a function of capacity *t*. The slope of the linear fit corresponds to κ. SRGAN results (κ≈2.78, red triangles) closely follow the MC benchmark (κ≈3.00, blue circles) and the Ising theoretical prediction (κ=3, dashed line). (**b**) Probability density function of the normalized driving function at fixed capacity t=1000. The histogram of SRGAN-generated data aligns well with the standard Gaussian distribution N(0,κ), confirming the Brownian motion nature of the driving function.

As shown in Figure 4, the SRGAN-generated configurations clearly reproduce the complex meandering features of Ising interfaces. To quantitatively verify physical fidelity, we extract interface coordinates from both SRGAN and MC configurations, calculating the driving function and capacity accumulation. We analyze the linear relationship between the driving function variance and capacity *t*, by setting a fixed capacity T=1000 and taking 20 equally spaced points in (0,T). As illustrated in Figure 5, the slope gives κ: the MC configuration yields κ≈3.000±0.004, while SRGAN yields κ≈2.784±0.0009.

Furthermore, examining the distribution of the normalized driving function $U_{t}/\sqrt{T}$ at T=1000 (Figure 5b), which should satisfy a normal distribution, the MC result gives κ≈2.979, while SRGAN gives κ≈2.761. Although the SRGAN value is slightly lower than the MC result (a deviation of about 5–8%), the physical order of magnitude remains consistent, indicating that the primary critical fluctuation modes have been captured.

To further validate the statistical properties of the generated interface, calculating the mean diffusion coefficient κ is insufficient; we must also verify whether the driving function increments strictly follow a Gaussian distribution. We employ the Kolmogorov–Smirnov (K-S) test to quantify the difference between the SRGAN-generated data distribution and the theoretical normal distribution. The K-S test evaluates goodness-of-fit by calculating the maximum vertical distance *D* between the empirical cumulative distribution function (ECDF) $F_{emp}\left(x\right)$ and the standard normal cumulative distribution function (CDF) $F_{theo}\left(x\right)$:

$$D=\underset{x}{\sup}\left|F_{emp}\left(x\right)-F_{theo}\left(x\right)\right|.$$

Smaller *D* values indicate a generated distribution closer to theory. To decouple the numerical deviation of κ from the verification of the distribution shape, we use the standardized variable $Z_{t}$. For a fixed capacity t=1000, we normalize the extracted driving function value $U_{t}$ by its sample standard deviation:

$$Z_{t}=\frac{U_{t}}{\sqrt{\mathrm{Var}(U_{t})}}.$$

We then test whether $Z_{t}$ follows a standard normal distribution N(0,1).

**Figure 6:**
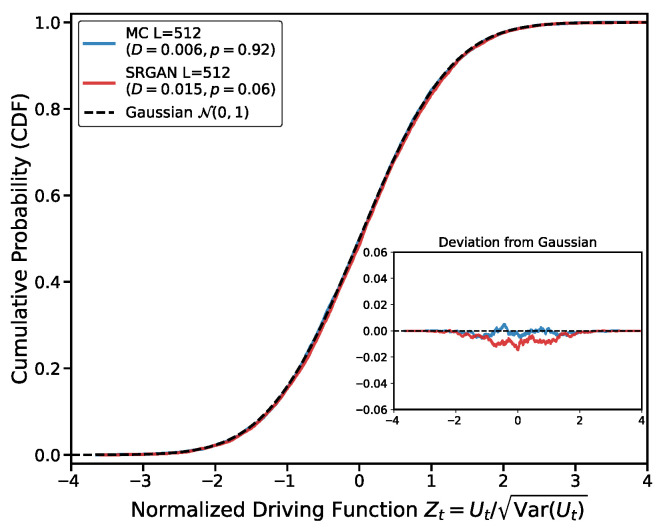
K-S test for the driving function increments. The main plot shows the Cumulative Distribution Function (CDF) of the standardized variable $Z_{t}$. SRGAN data (red line) overlaps highly with the theoretical standard normal distribution (black dashed line). Inset: Deviation between empirical and theoretical CDFs. The maximum deviation $D_{SRGAN}\approx 0.0149$, indicating that the generated interface retains the statistical properties of Brownian motion.

This method isolates the geometric shape characteristics of the distribution from κ estimation errors. The test is based on N=10,000 samples. As shown in Figure 6, the statistic for MC simulation data is $D_{MC}\approx 0.006$ (p>0.9), exhibiting perfect normality. For SRGAN-generated data, $D_{SRGAN}\approx 0.0149$ (p>0.05). The maximum deviation of the SRGAN-generated distribution from the normal distribution is only 1.49%. Additionally, the high overlap between the CDF curve and the Gaussian curve further confirms that SRGAN has successfully captured the Brownian motion statistical features of the driving function, reproducing the dynamics of the critical Ising model at the macroscopic scale. The shape of the distribution and linear variance of the driving function prove that the SRGAN-generated interface is not just visually similar, but dynamically reproduces the Markovian nature of the Ising interface. Through adversarial training, the network has captured the local stochastic laws governing interface growth.

### 3.2. Scale Generalization and Fractal Features

**Figure 7:**
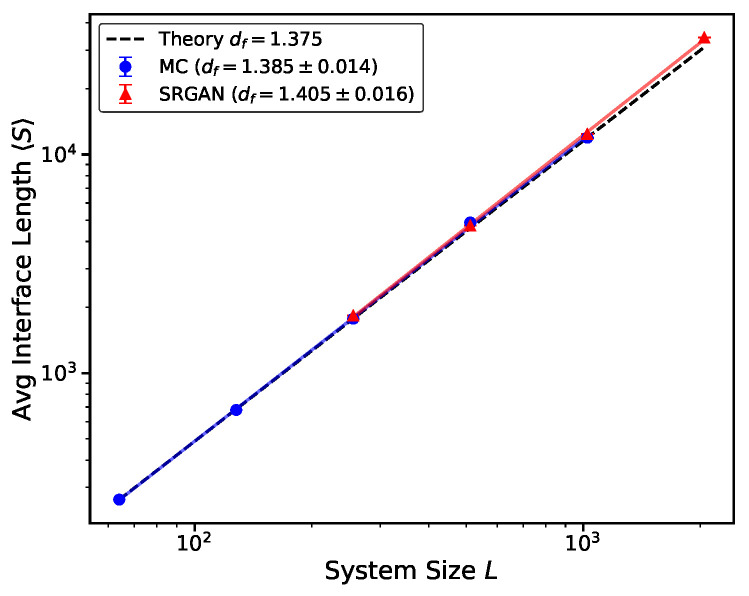
Finite-Size Scaling (FSS) analysis of fractal dimension $d_{f}$. Log–log plot of average interface length 〈S〉 versus system size *L*. Discrete points represent data for different sizes; solid lines represent power-law fits. MC configurations yield $d_{f}\approx 1.385$ (blue solid circles), while SRGAN-generated configurations yield $d_{f}\approx 1.405$ (red triangles).

The core advantage of SRGAN lies in its scale generalization capability. We input low-resolution sizes ($L_{\mathrm{LR}}=64,\;256,\;512$) not used in training into the model and successfully generate large-scale systems of ($L_{\mathrm{HR}}=256,\;1024,\;2048$) and then calculate the fractal dimension $d_{f}$ of interfaces at different sizes via Finite-Size Scaling analysis. As shown in Figure 7, the length *S* of the SRGAN-generated interface strictly follows the power law relation $S∼L^{d_{f}}$ with system size *L*. The fitted SRGAN fractal dimension is $d_{f}=1.405\pm 0.016$, which is very close to the theoretical value of the critical Ising model, $d_{f}=1.375$. Inferring κ from the SLE relation $d_{f}=1+\kappa /8$ yields κ≈3.24.

The measurement of fractal dimension $d_{f}$ reveals the model’s learning characteristics regarding microscopic geometric textures. The power-law growth of interface length confirms that the model has successfully captured the self-similarity of the critical state. However, the result of $d_{f}\approx 1.405$ is slightly higher than the theoretical 1.375.

This is not a failure of consistency but reflects a microscopic trade-off in the generation mechanism: to counter the smoothing tendency of the pixel-level loss function (L1), the random noise injected into the generator restores interface sharpness but inevitably introduces extra high-frequency roughness. This suggests SRGAN has found an equilibrium point—close to reality but slightly biased towards roughness—in its attempt to perfectly reproduce the geometric features of the system.

**Figure 8:**
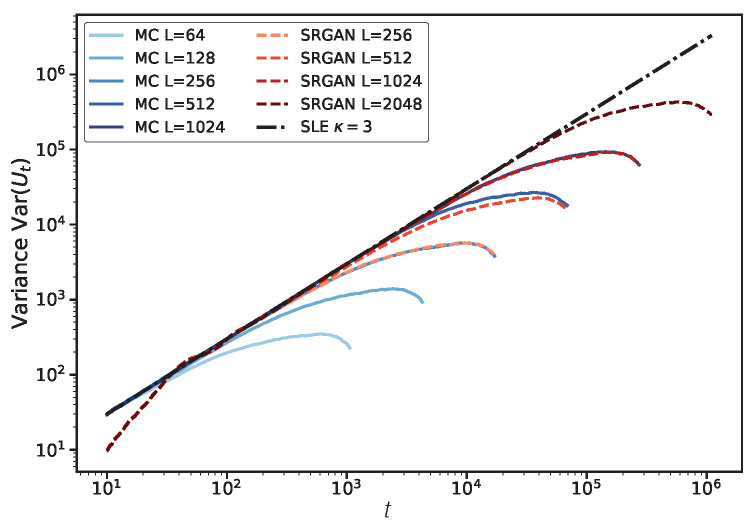
Verification of scale generalization capability. Comparison of driving function variance (colored dashed lines) for different sizes (L=256,…,2048) generated by SRGAN (trained only on 512×512) versus MC simulations. The model correctly reproduces the linear growth of variance on scales larger than the training set, as well as the bending phenomenon caused by finite-size boundary effects.

Next, we compare the statistical properties of the driving function $U_{t}$ across different scales. As shown in Figure 8, the SRGAN-generated interfaces match the overall dynamical behavior of MC simulations of the same size. Both exhibit clear finite-size effects: the growth rate of the driving function variance gradually decays as the evolution approaches the boundary. Notably, SRGAN results show a slight non-linear deviation at the beginning of evolution (t≈0), indicating the model is slightly less precise in capturing details at the starting boundary, though the reconstruction of the main interface remains accurate.

We speculate this might be related to the geometric augmentation strategies (rotation, flipping) used during training. Dobrushin boundary conditions are highly direction-sensitive; while data augmentation expands the sample size, it may introduce perturbations in the microscopic feature of how the interface initiates from the boundary, leading to transient statistical deviations in the initial stage of evolution.

**Figure 9:**
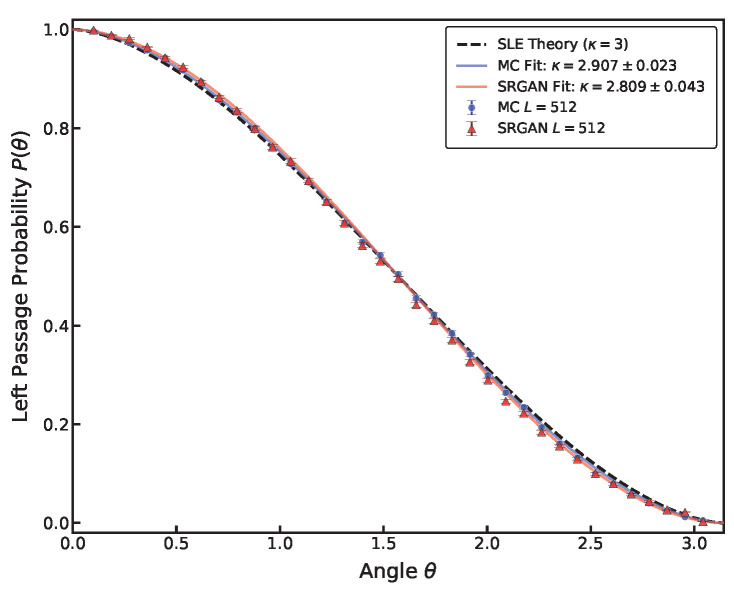
Testing conformal invariance via Left Passage Probability (LPP). The plot shows the empirical probability P(θ) of the interface passing to the left of a point with polar angle θ in the upper half-plane. SRGAN results (red triangles) align very closely with Schramm’s formula for κ=3 (black solid line), confirming that global topological properties of the critical interface are preserved during super-resolution.

**Figure 10:**
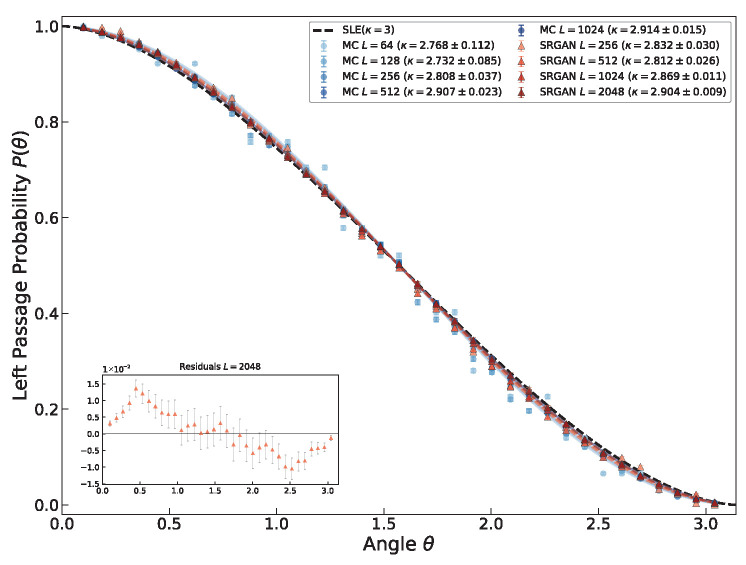
Verification of scale invariance in Left Passage Probability (LPP). Empirical LPP distributions P(θ) are plotted for different system sizes, ranging from small MC simulations (L=64, light blue circles) to massive SRGAN-generated configurations (L=2048, dark red triangles). The bottom left figure illustrates the residuals of the L=2048 SRGAN-generated configurations. Despite the vast difference in lattice dimensions, all datasets collapse onto the single theoretical curve predicted by ${\mathrm{SLE}}_{3}$ (black solid line). This collapse confirms that SRGAN-generated interfaces strictly obey the conformal scale invariance required by critical fixed points, preserving geometric universality across scales.

For an SLE_*κ*_ curve in the upper half-plane H starting from the origin, the Left Passage Probability P(θ) is defined as the probability that the curve passes to the left of a reference point $z=re^{i\theta }$. By conformal invariance, this probability depends only on the polar angle θ∈(0,π) and is independent of the modulus *r* [40], as given by Schramm’s formula (Equation (7)). As shown in Figure 9 and Figure 10, both MC configurations (κ≈2.907) and SRGAN-generated configurations (κ≈2.869) yield results close to those calculated from the linear relationship between driving function and capacity. Furthermore, we have calculated the LPP for configurations of other sizes. As shown in Figure 10, results for both MC and SRGAN configurations collapse remarkably well onto the theoretical curve of ${\mathrm{SLE}}_{3}$. This confirms that SRGAN has learned the global topological constraints. Although microscopic textures may be affected by noise, on the macroscopic scale, the generated interfaces strictly follow the rotational and scale invariance required by Conformal Field Theory (CFT). This proves the neural network has successfully encoded the overall geometric feature of the critical state.

## 4. Discussion

Our findings demonstrate that SRGAN can reproduce the critical geometric features of large-scale Ising configurations. Through rigorous SLE theory testing, we find the diffusion coefficient κ, fractal dimension $d_{f}$, and Left Passage Probability $P_{LPP}$ of the generated interfaces all approximate theoretical predictions (${\mathrm{SLE}}_{3}$).

Near the critical point, the Ising model possesses scale invariance. Traditional super-resolution techniques aim to restore high-frequency textures in images; in the Ising model, these textures correspond to short-range spin fluctuations. Our model, trained on a 128→512 mapping, successfully generalizes to 512→2048.

To test whether the network has transcended local texture synthesis, we examine the two-point spin correlation function, defined as $G\left(r\right)={\langle \sigma_{i}\sigma_{j}\rangle }_{|r_{ij}|=r}$. Physically, it quantifies how strongly the state of one spin influences another at a distance *r*. While correlations in off-critical phases decay exponentially (indicating finite correlation length), the critical state is uniquely characterized by a heavy-tailed power-law decay:

(8) $$G\left(r\right)∼r^{-\eta },$$

where η=1/4 is the critical exponent for the 2D Ising model.

**Figure 11:**
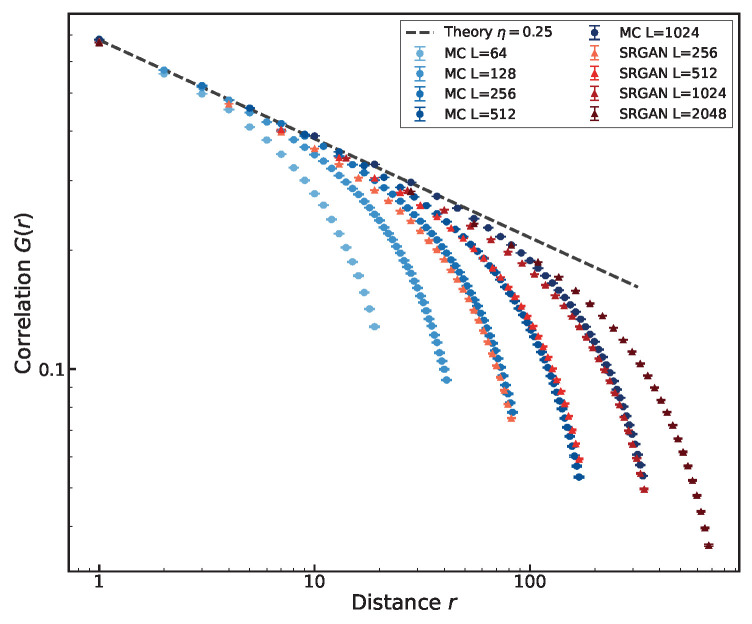
Two-point correlation function G(r). Results show that SRGAN-generated configurations retain the correct power-law correlations over long distances.

Figure 11, displaying the two-point correlation function G(r), supports this view: configurations of all sizes follow a power-law decay with η≈0.25. This decay behavior persists over distances far exceeding the training size, vanishing only when the power-law decay reaches half the lattice size due to the interface. This indicates SRGAN retains the physical correlation information of the critical Ising model, rather than merely performing local texture synthesis. The power-law behavior of G(r) confirms the model captures long-range physical correlations. SRGAN effectively executes an implicit inverse coarse-graining transformation: it is not merely interpolating pixels, but reconstructing the short-range fluctuations.

Regarding the κ value deviation found in the SLE analysis (driving function variance gives κ≈2.78, fractal dimension gives κ≈3.24), this is not a simple error but reflects a tug-of-war between physical constraints and stochasticity during training:

- Origin of Low κ (Smoothing Effect): To maintain spin discreteness and domain wall sharpness, we employed L1 loss. As a strong supervisory signal, L1 forces the network to output a statistically averaged path when facing random thermal fluctuations, suppressing large meanders of the interface at long-wave scales, leading to a lower macroscopic κ.
- Origin of High $d_{f}$ (Roughness Effect): To prevent mode collapse, we injected random noise into the generator. This restores microscopic diversity but introduces extra high-frequency roughness. Since fractal dimension is highly sensitive to microscopic details, this leads to an inflated measurement.

The success of SRGAN is no accident. The Ising model is defined on a regular Euclidean grid, and its Hamiltonian possesses strict translational symmetry. The fully convolutional architecture (FCN) we adopted enforces translational equivariance, constituting an inductive bias that matches the physical system [20]. This explains why the model only needs to learn local spin interaction patterns to generalize to systems of arbitrary size—because local physical laws do not change with system size.

In addition, SRGAN provides a computationally accessible route to large-scale critical configurations. Generating 10,000 equilibrium configurations at L=512 via parallel Monte Carlo simulation (20 CPU cores) requires approximately 25 h. By contrast, the SRGAN first generates 10,000 low-resolution configurations at L=128 via Monte Carlo (approximately 2.11 h), then upsamples these to L=512 using the trained generator (approximately 1 min), reducing the total cost to approximately 2.13 h. The one-time training cost of 7.7 h is amortized over all subsequent inference runs. Furthermore, the fully convolutional architecture generalizes across scales without retraining: the same model can upsample L=512 inputs to L=2048, producing 10,000 configurations in approximately 800 s, a scale at which direct Monte Carlo simulation becomes prohibitively expensive.

## 5. Conclusions

This paper maps the generative capability of neural networks onto a concrete physical landscape, by integrating the multi-dimensional analysis of SLE theory.

At the level of global statistical structure, the model has internalized the statistical skeleton of the critical state. The long-range power-law decay of the two-point correlation function G(r) and the scale collapse of the LPP confirm that SRGAN is not simply synthesizing textures. Instead, it accurately captures the long-range physical correlations and global conformal invariance governing the critical system. This means the generated configurations possess high physical fidelity in topological structure and long-wave fluctuations.

At the level of interface dynamics, the Gaussian distribution of driving function increments and the linear growth of variance indicate that SRGAN has learned the local Markovian nature of interface growth, successfully reproducing the stochastic evolution driven by Brownian motion. Although the diffusion coefficient κ shows a slight numerical underestimation, this reflects a difference in diffusion rate without altering the essential random walk nature of the motion.

The deviation in geometric features reveals a trade-off between clarity and precision. The scaling law of the fractal dimension $d_{f}$ verifies the self-similarity of the interface, yet its slight numerical overestimation (κ≈3.24 from $d_{f}$) contrasts with the underestimation from driving function variance (κ≈2.78). This deviation profoundly reflects the balancing mechanism of the loss function: the L1 loss attempts to smooth the interface to reduce error (lowering κ), while noise injection attempts to add detail to maintain sharpness (raising $d_{f}$). It is within this dynamic equilibrium that SRGAN generates critical interfaces that are statistically self-consistent, albeit slightly rough in microscopic detail.

The deviation of approximately 7% in the diffusion coefficient indicates that SRGAN is not yet a substitute for Monte Carlo simulations where high-precision estimation of critical exponents is required. Nevertheless, it establishes a new computational paradigm for the study of large-scale critical phenomena, offering a low-cost route to generating physically faithful configurations at scales inaccessible to direct simulation. The present framework is in principle extensible to other universality classes whose critical interfaces admit an SLE description, such as the q-state Potts model, critical percolation, and the self-avoiding walk, provided that specific training data are available. Future work could further close the precision gap by refining the loss landscape, incorporating physical constraints directly into the training objective, or exploring probabilistic generative approaches such as diffusion models, which may offer a more natural inductive bias for reproducing the stochastic geometry of critical interfaces.

## Data Availability

All code is implemented in Python 3.12 (Ubuntu 22.04). Deep learning models are implemented and trained using PyTorch 2.7.0 with CUDA 12.8 on a single NVIDIA RTX 5090 GPU (32 GB VRAM). Readers requiring access to the raw data/code may contact the first author, who will provide it via cloud storage upon reasonable request.
